# Multi-Morbidity and Polypharmacy in Older People: Challenges and Opportunities for Clinical Practice

**DOI:** 10.3390/geriatrics5040085

**Published:** 2020-10-28

**Authors:** Pritti Aggarwal, Stephen J. Woolford, Harnish P. Patel

**Affiliations:** 1 Southampton City Clinical Commissioning Group, Southampton SO16 4GX, UK; prittiaggarwal@nhs.net; 2 Living Well Partnership, Southampton SO19 9GH, UK; 3 School of Primary Care, Population Sciences and Medical Education, University of Southampton, Southampton SO17 1BJ, UK; 4 Medicine for Older People, University Hospital Southampton NHS FT, Southampton SO16 6YD, UK; stephen.woolford@uhs.nhs.uk; 5 Academic Geriatric Medicine, University of Southampton, Southampton SO16 6YD, UK; 6 NIHR Biomedical Research Centre, University of Southampton and University Hospital Southampton NHS FT, Southampton SO16 6YD, UK

**Keywords:** multi-morbidity, polypharmacy, comprehensive geriatric assessment, age-friendly care, patient priority care, frailty, deprescribing

## Abstract

Multi-morbidity and polypharmacy are common in older people and pose a challenge for health and social care systems, especially in the context of global population ageing. They are complex and interrelated concepts in the care of older people that require early detection and patient-centred shared decision making underpinned by multi-disciplinary team-led comprehensive geriatric assessment (CGA) across all health and social care settings. Personalised care plans need to remain responsive and adaptable to the needs and wishes of the patient, enabling the individual to maintain their independence. In this review, we aim to give an up-to-date account of the recognition and management of multi-morbidity and polypharmacy in the older person.

## 1. Introduction

Globally, the number of people aged 60 or over is set to rise from 841 million to more than 2 billion between 2013 and 2050; this equates to 21.1% of the world’s population [1]. The proportion of people aged 80 years or over is growing even faster; estimated to be 125 million in 2015 contrasted to 71 million worldwide at the turn of the millennium. This number is projected to increase by 61 per cent over the next 15 years, reaching nearly 202 million in 2030 [2]. In the United Kingdom (UK), a male aged 85 could expect to live to age 90.8 years and a female to 91.8 years (Office of National Statistics 2016, ONS.gov.uk). These demographic changes are largely due to the successful advances in public health and modern medicine that have allowed people to live with one or more long-term conditions. This is undoubtedly a cause for celebration, but the challenge posed by cumulative demographic changes of such magnitude require immediate action from multi-disciplinary teams to provide patient-centred and age-friendly health and social care [3,4].

In this narrative review of the literature, we aim to give an up-to-date account on the definitions and recognition of multi-morbidity and polypharmacy as well as offer insights into models of care that have been shown to be effective and that health care professionals involved in the care of older people across primary and secondary care can readily implement in clinical practice. 

## 2. Methods

A literature review was carried out utilising medical journal databases, including PubMed and the Cochrane Library. Search terms pertinent to this topic used were “multi-morbidity”, “polypharmacy”, “deprescribing”, “comprehensive geriatric assessment”, “age friendly care”, and “patient priority care”. Article titles and abstracts were then assessed for relevance and full-text screening performed if the title or abstract contained one or more search terms and the article itself was deemed relevant to this topic. Articles were also selected for further review from the wider literature based on the authors own clinical expertise and knowledge of pre-existing work in this field. Only English language articles published between 2003 and 2020 were included (Appendix A). We applied the principles from the Scale for the Assessment of Narrative Review Articles (SANRA) to this manuscript [5] (see Appendix B). 

### 2.1. Multi-Morbidity

Multi-morbidity refers to the presence of two or more simultaneous long-term health conditions in the older individual concerned [4]. These can span physical as well as psychosocial domains and include conditions such as cardiovascular, metabolic, musculoskeletal disease, mental health illness, chronic pain, sensory deprivation, and substance misuse [6,7]. Multi-morbidity increases with age but is not only limited to older people. For example, in a cross sectional study of over 1 million patients in Scotland, the prevalence of multi-morbidity was 30.4% in those aged 45–64 years increasing to 81.5% in those >85 years [6]. A higher prevalence of multi-morbidity is present in women and those who have a lower socio-economic status and educational attainment. In addition, there are racial and ethnic differences that affect prevalence rates [4,8]. 

Long-term health conditions account for approximately 50% of general practitioner (GP) appointments, 64% of hospital outpatient appointments, and 70% of inpatient hospital admissions [8] and is therefore responsible for approximately 70% of the United Kingdom, National Health Service (UK NHS) current healthcare expenditure. People living with multi-morbidity are at greater risk of incident as well as worsening of pre-existing mental health problems, unplanned hospital admission, experience higher rates of polypharmacy and adverse drug reactions (ADR), and have a reduced quality of life [9,10,11,12]. Living with multi-morbidity is associated with increased rates of mortality. For example, adults aged >60 years with ≥2 or ≥3 health conditions had a 1.73 (95% CI: 1.41; 2.13) and 2.72 (95% CI: 1.81; 4.08) increased risk of dying respectively, compared to those who were not multi-morbid [13]. The proportion of patients living with multi-morbidity is likely to climb as life expectancy continues to rise [6] and is fast becoming a global health problem [14,15,16].

The associations between multi-morbidity and poor outcome are likely to be multifactorial. Health conditions have a tendency to cluster and interact with other related diseases and can worsen the severity of each disease or result in the development of a further potentially more serious condition [17]. For example, the metabolic syndrome is characterised by central obesity alongside the presence of insulin resistance, hypertension, and hyperlipidaemia [18]. Interaction between these diseases leads to a worsening of each individual condition, as well as an increased risk of cardiovascular events such as stroke or myocardial infarction [19,20]. Further examples relevant for older people are congestive cardiac failure and dementia—where individuals often have several co-existent long-term conditions such as diabetes and hypertension and have impairments in activities of daily living (ADL) and physical function [21]. Patients such as these who are multi-morbid and have functional limitations can experience the poorest health outcome from the inability to self-manage chronic long-term conditions as well as the ensuing polypharmacy [22].

In clinical practice, healthcare infrastructure has not been optimised to manage multiple diseases simultaneously, which leads to disorganized care for those who are multi-morbid. The norm amongst most medical professionals out with geriatric medicine is to specialise and manage single organ systems, or even a single disease within a given specialty. Whilst this is sometimes necessary, treating diseases in isolation can lead to the duplication of efforts as well as fragmented, poorly coordinated healthcare assessments without adequate responsibility, accountability, and follow up [23,24]. The emphasis on managing single diseases in isolation ignores the unique dynamics of disease clusters. Furthermore, clinical trials of new medications often exclude participants who have an additional health problem to the one being investigated [25,26,27] and importantly, often by design, exclude older people [28,29,30]. This causes uncertainty regarding the potential risks and benefits of starting evidence-based medications for an older patient in the context of their multi-morbidity and invariably leads to additional treatment burden, lower adherence, and increased risk of ADR [31].

### 2.2. Multi-Morbidity and Frailty

Multi-morbidity and frailty are associated conditions, but the terms should not be used interchangeably as often is the case amongst specialties in the author’s clinical experience. A recent meta-analysis of 78,122 participants of 48 studies found that, on average, seven out of ten adults living with frailty were also multi-morbid [32]. However, people living with multi-morbidity were not necessarily found to be living with frailty, as many people with multiple health conditions can have the sufficient physiological reserve to recover from insults, restore homeostasis, and return to their previous baseline of health. 

### 2.3. Polypharmacy

Polypharmacy, defined as the concurrent use of at least 4–5 medications rises considerably as the number of health problems and healthcare service use increases [33,34]. In the health survey for England 2016, 56% of individuals aged 85 and over were taking five or more medicines compared to 9% of those aged 45–54. A study of 180,815 primary care records found that amongst patients with two comorbidities, 20.8% received four to nine medications and 1.1% received ten or more medications [35]. In contrast, amongst patients with six or more comorbidities, these values increased to 47.7% and 41.7%, respectively. Several studies have also found that the number of medications increased after hospital admission with the prescription of two additional drugs on average [36,37].

In the past decade, the average number of items prescribed for each person per year in England has increased. For example, the proportion of patients receiving ≥10 medications was 1.9% in 1995, increasing to 5.8% in 2010 [33]. One explanation for this rise is that asymptomatic people are increasingly treated with preventative interventions to reduce their future risk of mortality; this is seen particularly with cardiovascular disease. If each co-morbid condition is treated in accordance to national guidelines, patients would be on many more medications [38]. The absolute benefit offered by each additional medicine is likely to reduce when a person is taking multiple preventative medicines. In addition, the risk of harm is likely to increase the more medicines a person takes. However, not all polypharmacy is inappropriate. Prescriptions are appropriate in instances where medicines have been optimised for complex conditions according to best evidence. Advanced age in itself should not be a reason for withholding effective therapies [39]. Problematic polypharmacy occurs when there is the prescription of multiple medicines and the risk of harm outweighs benefits and the consequent pill burden leads to lower adherence, ADR or risks of potentially harmful interactions. ADR can lead to further morbidity. For example, within two hospitals in the UK, a study suggested that the prevalence of ADR-related admissions was 6.5%, with ADR directly leading to acute admission in 80% of cases [40]. In a follow up study of hospital wards, it was estimated that one in seven patients experienced an ADR that contributed to health deterioration and increased the length of hospital stay [41]. 

### 2.4. Management of Multi-Morbidity and Polypharmacy

Key points to managing older individuals living with multi-morbidity and polypharmacy include early identification of those living with multiple health conditions, identification of frailty, and patient-centred shared decision making [42,43,44,45]. Underpinning these points are the 5 tenets of age-friendly care: what matters most to the patient; multi-complexity management, including psychosocial problems; medication management; mentation—to account for cognitive awareness when making shared decisions; and mobility—to account for gait and balance problems when considering holistic management of the patient [24,46,47]. This tool allows clinicians to capture and communicate complexities in understandable and easily remembered terms. Poor communication between clinicians as well as between clinicians and patients/caregivers is the most common cause of adverse clinical events and complaints about care. In this regard, patient-centred or patient priority care that accounts for the patient’s preferences, needs, and values ensures that patients or their appointed attorneys guide clinical decision making [48]. 

Balancing the recommendations of multiple guidelines for those who are multi-morbid inevitably leads to polypharmacy and danger of the prescribing cascade where medications are prescribed to counter side-effects of another medication. Medicine optimisation is defined as a person-centred, evidence-based approach to safe and effective medicine use to ensure people obtain the best possible outcomes from their medicines and that they continue to provide benefit for the individual. Medicines optimisation ensures that there is a specific and justifiable reason for every medication the patient is taking and that this is as optimum as it can be based on evidence. This includes stopping medications that are having no benefit or causing side effects, interacting and/or are contra-indicated but also upgrading prescriptions to newer medications according to contemporaneous evidence and guidance. Several guides are available to help with medicines optimisation such as the Beers Criteria, Medications Appropriateness Index, STOPP-START, NO TEARS, and the PINCER tool [49,50,51,52]. These guides rely on actions taken by all health and social care practitioners and requires patient engagement and professional collaboration across health and social care settings [53,54,55]. 

Medicine optimisation in older people is especially important for drugs with a narrow therapeutic index (NTI) such as digoxin, warfarin, aminophylline, lithium, and some antidepressants. Older people are more susceptible to anticholinergic side effects from commonly prescribed drugs such as amitriptyline, oxybutynin, cetirizine, and mirtazapine, which include delirium, reduced cognition, gait and balance problems, constipation, urinary retention, and dry mouth [56]. These conditions may be misinterpreted as new conditions and can initiate a prescribing cascade. In addition, older individuals who have a shorter life expectancy may not benefit from prescribed medications that take time before therapeutic benefit is established. Examples include anti-hypertensives drugs used to treat hyperlipidaemia and osteoporosis treatments [57,58]. 

Deprescribing requires knowledge of the potential for ADR as well as patient factors spanning physical, social, and psychological domains, e.g., cognition and physical capability. When planned, patient-centred, and backed up by education and training, deprescribing has not been associated with any significant side effects or adverse outcomes [59,60,61]. Older individuals frequently dislike taking multiple prescribed medications for several reasons such as complex dosing regimens, fear, scepticism—especially when asymptomatic—and media portrayal. These factors affect adherence, and studies suggest that up to 50% of prescribed medications may not be taken by older people [62]. Given the heterogeneity of disease trajectories in older people as well as symptoms and individual patient preferences, goals of care will vary between individuals. GPs are in an ideal position to make shared decisions with patients and families to prescribe, deprescribe, rationalise, and optimise medications but need funded time and space to nurture long-lasting, trustful therapeutic partnerships [53,63,64]. It is also clear that the impact of multi-morbidity, frailty, and polypharmacy spans both primary and secondary care. Geriatricians and pharmacists are ideally placed alongside GPs to aid goal setting, implement the principles of comprehensive geriatric assessment (CGA), and provide patient-centred care across this arbitrary primary/secondary care divide [65,66,67,68]. 

**Figure 1:**
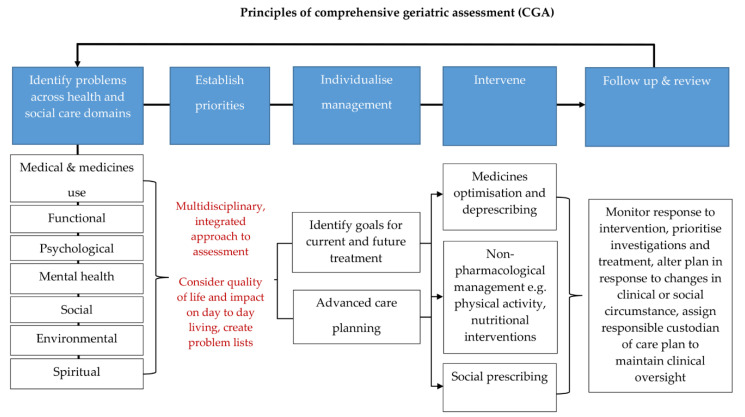
Principles of comprehensive geriatric assessment (CGA). CGA is an evidence-based multidimensional and interdisciplinary assessment of medical, psychological, and functional capabilities aimed at developing an integrated plan for treatment and care. CGA is associated with favourable clinical and health care outcomes. The core principles of CGA include comprehensive history taking and multidisciplinary led assessment, culminating in goals for current and future management. They encompass the 5 tenets of age-friendly care: 1. what matters most to the patient, 2. multi-complexity management, 3. medication management, 4. mentation, and 5. mobility. These principles can be applied across any health and social care setting and have been shown to be highly effective in the management of older people living with frailty and multi-morbidity. The process is iterative and the key to its success is timely review and coordination so that the care plan generated from a CGA remains responsive to the patient’s needs [24,46,66,69,70].

Patient-centred, age-friendly care and polypharmacy management are embodied in the core principles of CGA where a holistic and balanced approach to individualise and prioritise management opinions take centre stage. Exploring a person’s personal goals, the health problems that have the most impact on their day-to-day life and choice on their medication regime allows a tailored approach, which can facilitate an improvement in individual quality of life. This positive approach, inclusive for all individuals irrespective of their capacity and capability, is summarised in Figure 1. 

## 3. Conclusions

Older age is associated with an increased risk of accumulating multiple long-term conditions. Multi-morbidity and polypharmacy are associated with progressive loss of resilience and impaired homeostasis, that contribute to significant health and social care burden. Implementing an age-friendly care paradigm where there is routine assessment of long-term conditions, ascertainment of frailty, and medicines optimisation should be the goal for clinicians across all health and social care settings. 

## Figures and Tables

**Figure A1:**
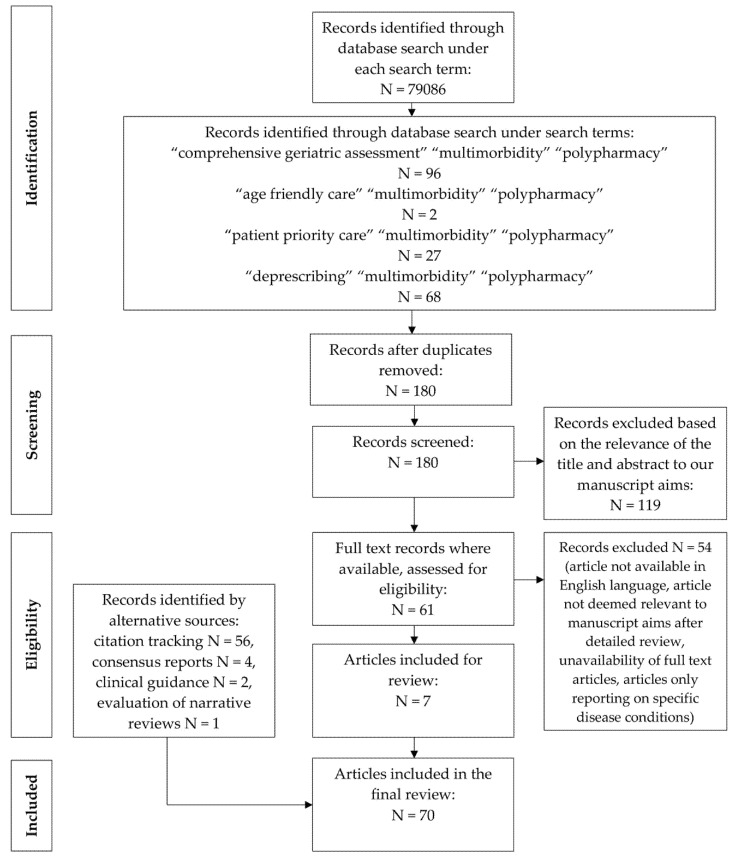
Flow chart for article selection.

## Appendix A

**Search Strategy:** A literature review was carried out utilising medical journal databases, including PubMed and the Cochrane Library. Search terms pertinent to this topic used were “multi-morbidity”, “polypharmacy”, “deprescribing”, “comprehensive geriatric assessment”, “age friendly care”, and “patient priority care”. Article titles and abstracts were then assessed for relevance and full-text screening performed if the title or abstract contained one or more search terms, and the article itself was deemed relevant to this topic. Articles were also selected for further review from the wider literature based on the authors own clinical expertise and knowledge of pre-existing work in this field. Only English articles language published between 2003 and 2020 were included. We applied the principles from the Scale for the Assessment of Narrative Review Articles (SANRA) to this manuscript.

## Appendix B

**Table A1:** Scale for the Assessment of Narrative Review Articles (SANRA). We rated the content of this manuscript according to the Scale for the Assessment of Narrative Review Articles (SANRA) [5]. The sum score was 10 out of a maximum of 12 points indicating a high-quality manuscript.

**1**	**Justification of the article’s importance for the readership**		
	The importance is not justified	0	
	The importance is alluded to, but not explicitly justified	1	
	The importance is explicitly justified	2	X
**2**	**Statement of concrete aims or formulation of questions**		
	No aims of questions are formulated	0	
	Aims are formulated generally but not concretely or in terms of clear questions	1	
	One or more concrete aims or questions are formulated	2	X
**3**	**Description of literature search**		
	The search strategy is not presented	0	
	The literature search is described briefly	1	X
	The literature search is described in detail, including search terms and inclusion criteria	2	
**4**	**Referencing**		
	Key statements are not supported by references	0	
	The referencing of key statements is inconsistent	1	
	Key statements are supported by references	2	X
**5**	**Scientific reasoning (e.g., incorporation of appropriate evidence, such as RCTs in clinical medicine**		
	The article’s point is not based on appropriate arguments	0	
	Appropriate evidence is introduced selectively	1	
	Appropriate evidence is generally present	2	X
**6**	**Appropriate presentation of data (e.g., absolute vs. relative risk; effect sizes without confidence intervals)**		
	Data are presented inadequately	0	
	Data are often presented in the most appropriate way	1	X
	Relevant outcome data are generally presented appropriately	2	
	**Sum score**	**10**	

RCTs: Randomised Controlled Trials.
